# Effects of REDOX in Regulating and Treatment of Metabolic and Inflammatory Cardiovascular Diseases

**DOI:** 10.1155/2020/5860356

**Published:** 2020-11-17

**Authors:** Kai Wang, Yanhan Dong, Jing Liu, Lili Qian, Tao Wang, Xiangqian Gao, Kun Wang, Luyu Zhou

**Affiliations:** Institute of translational medicine, The Affiliated Hospital of Qingdao University, Qingdao University, Qingdao 266021, China

## Abstract

Reduction oxidation (REDOX) reaction is crucial in life activities, and its dynamic balance is regulated by ROS. Reactive oxygen species (ROS) is associated with a variety of metabolic diseases involving in multiple cellular signalling in pathologic and physiological signal transduction. ROS are the by-products of numerous enzymatic reactions in various cell compartments, including the cytoplasm, cell membrane, endoplasmic reticulum (ER), mitochondria, and peroxisome. ROS signalling is not only involved in normal physiological processes but also causes metabolic dysfunction and maladaptive responses to inflammatory signals, which depends on the cell type or tissue environment. Excess oxidants are able to alter the normal structure and function of DNA, lipids, and proteins, leading to mutations or oxidative damage. Therefore, excessive oxidative stress is usually regarded as the cause of various pathological conditions, such as cancer, neurodegeneration, cardiovascular diseases (CVDs), diabetes, and kidney diseases. Currently, it has been possible to detect diabetes and other cardiac diseases by detecting derivatives accompanied by oxidative stress in vivo as biomarkers, but there is no effective method to treat these diseases. In consequence, it is essential for us to seek new therapy targeting these diseases through understanding the role of ROS signalling in regulating metabolic activity, inflammatory activation, and cardiac diseases related to metabolic dysfunction. In this review, we summarize the current literature on REDOX and its role in the regulation of cardiac metabolism and inflammation, focusing on ROS, local REDOX signalling pathways, and other mechanisms.

## 1. Introduction

Oxidative stress can be defined as active oxygen/nitrogen excessive production of ROS, such as oxidant, and lack of antioxidant enzymes. The detoxification of compounds in the cells is usually normal, but when the oxidant emissions are excessive, the cell produces excessive oxidation material to change DNA lipid and protein structure, leading to cell mutation and oxidative damage. Excessive oxidative stress, therefore, is considered as the causes and consequences of a variety of pathological processes, including cancer, neural degeneration, CVDs, diabetes, and kidney diseases [1, 2]. Some studies have found that a balance of oxidative stress is associated with aging [3]. Most kinds of natural or synthetic antioxidants have been evaluated against the oxidative stress-related pathological changes [1, 4, 5]. Besides, ROS is a by-product of many cell compartment enzymatic reactions occurring on the cytoplasm membrane endoplasmic reticulum (ER), or mitochondria, which can control intracellular environment balance and work as the main regulatory factor of cell dysfunction in the pathophysiology. In different cell types or organizational environment, ROS signals may participate in increased inflammatory of incommensurate reaction or lead to metabolic dysfunction-related diseases, such as atherosclerosis, diabetes, and heart stroke [6]. In addition, emerging studies have revealed that a healthy diet plays a critical role in the prevention of CVDs by modulating the oxidative balance [7, 8]. For example, a healthy diet can prevent atherosclerosis by inhibiting the oxidation of low-density lipoprotein (LDL) and reducing the production of ROS [9]; the results of the PREDIMED study [10] show that highly unsaturated fat and antioxidant-rich dietary patterns are useful for reducing the risk of CVDs. Therefore, understanding of ROS signals in regulating metabolic activity and inflammation will promote the discovery of new therapies treating CVDs.

## 2. ROS Generation

**Figure 1:**
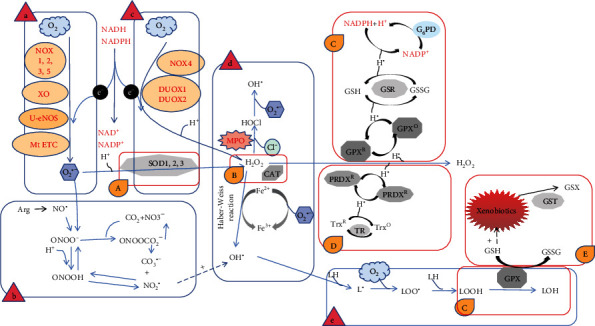
ROS generation and clearance. I. ROS generation: (a) superoxide formation; (b) reactive nitrogen species (RNS) formation; (c) hydrogen peroxide formation; (d) hydroxyl radical formation; (e) lipid radical formation. II. ROS clearance: (A) superoxide dismutation; (B) hydrogen peroxide decomposition; (C) glutathione redox cycle; (D) thioredoxin redox cycle; (E) glutathione-*S*-transferase detoxification.

The generation of ROS is involved in a series of complex biochemical reactions [11]. The cascade of ROS generation consists of the following five main pathways (Figure 1):

- *O*_2^−^_*formation*: O_2_− is produced by the coupling of O_2_ with electrons (e^−^) from donors, which is usually considered to be the first ROS cascade reaction. In mammalian cells, e^−^ donors are usually reduced nicotinamide adenine dinucleotide (NADH) or reduced nicotinamide adenine dinucleotide phosphate (NADPH). O_2_− can be converted into other kinds of ROS by an oxidation reaction.
- *RNS formation*: RNS is a derivative of NO∙, and NO∙ is produced by L-arginine (L-Arg) and catalyzed by NOS. NO∙ can react quickly with O_2_∙− to form ONOO^−^. The second-order rate constant between NO∙ and O_2_∙− is nearly 10 times faster than that of O_2_∙− catalyzed by superoxide dismutase [12, 13]. However, due to the high intracellular SOD content under physiological condition, O_2_− was removed before encountering NO∙.
- *H_2_O_2_ formation*: H_2_O_2_ is produced by O_2_− mutation catalyzed by superoxide dismutase (SOD). At low pH, a small amount of O_2_− mutation occurs spontaneously, and some of which can react with reductive transition metals, such as [4Fe-4S]^2+^. Some oxidases (e.g., NOX4 and DUOX1/2) have dismutase activity and can directly convert O_2_ into H_2_O_2_ instead of O_2_−.
- *OH∙ formation*: OH∙ can be generated from homolysis fission of ONOOH, and most of OH∙ is formed by metal ions (iron or copper) catalyzed by H_2_O_2_ and O_2_− through the Habor-Weiss reaction. In diseases with iron accumulation (e.g., atherosclerotic lesions [14] or sickle cell patients [11]), OH∙ mediated oxidative stress might be the most pivotal mechanism. OH∙ has strong oxidation ability and short half-life, which is the major cause of biological macromolecule damage by ROS.
- *L*∙*/LOO*∙ *formation*: The highly active OH∙ or ONOO^−^ can react with the polyunsaturated fatty acid (PUFA) of the biofilm for lipid peroxidation, in which OH∙ can react directly with lipids to capture a hydrogen atom to form a carbon-centered lipid free radical (L∙). L∙ initiates lipid peroxidation under an aerobic condition and generates lipid peroxide group (LOO∙), which is a medium oxidant that can extract H from nearby lipids to produce lipid hydrogen peroxide (LOOH). Moreover, L∙/LOO∙ can exist in the reaction process of the lipoxygenase-catalyzed polyunsaturated fatty acid formation of molecular oxygen to form hydroperoxide [15, 16].

## 3. Dynamics of ROS

In order to maintain the stability of ROS, there are five active oxygen scavenging pathways:

- O_2_− mutated to H_2_O_2_ by superoxide dismutase (SOD)
- Catalase (CAT) decomposes H_2_O_2_ to produce H_2_O and O_2_
- *Glutathione redox cycle*: using glutathione as an electron donor, H_2_O_2_ and LOOH are decomposed by glutathione peroxidase (GPX).
- *Thioredoxin reduction cycle*: using reduced thioredoxin (TrxR) as electron donor, H_2_O_2_ was reduced by redox protein (PRDX) 1-5 to produce H_2_O.
- Exogenous detoxification of glutathione transferase (GST)

## 4. Oxidative Stress and CVDs

CVD is the leading cause of death worldwide [17], which is a complex pathophysiological disease involved in many factors. The dysdynamics of ROS has been regarded as one of the potential pathogenic factors [18–20]. Increased ROS level is able to lead to decreased availability of nitric oxide and vasoconstriction, which subsequently promotes arterial hypertension [21]. ROS also has negative effects on myocardial calcium treatment, inducing arrhythmias and cardiac remodeling by facilitating hypertrophic signal transduction and apoptosis [22, 23]. In addition, it also promotes the formation of atherosclerotic plaques [24].

### 4.1. Arterial Hypertension

It is estimated that the global prevalence of hypertension was 1.13 billion in 2015, the risk of which becomes higher with age [25]. A large number of studies have shown that ROS plays an important role in the pathogenesis of hypertension [26–28].

In the vascular system, ROS is mainly produced by vascular endothelial cells, adventitia cells, and smooth muscle cells, primarily induced by NADPH oxidase which produces O_2_− upon being stimulated by Angiotensin II (Ang-II), Endothelin-1 (ET-1), or urotensin II (U-II). On the other side, increased mechanical forces caused by elevated blood pressure, such as unidirectional laminar flow and oscillatory shear stress, can help to increase the accumulation of ROS. Ca^2+^ is involved in the regulation of cell contraction, secretion, metabolism, gene expression, and cell survival [29]. The interaction of ROS and Ca^2+^ plays an important role in the occurrence and development of CVDs [30–32]. Store-operated Ca^2+^ channel (SOCC) is a ubiquitous Ca^2+^ influx pathway and is the dominant Ca^2+^ channel in unexcited cells [33, 34]. ORAI/STIM is a highly selective calcium channel and an important component of SOCC [35, 36]. ORAI/STIM channel is involved in a variety of cardiovascular physiological processes [37]. Oxidative stress can regulate the activity of the ORAI/STIM channel by uncoupling ORAI/STIM complex, regulating the gene expression of ORAI or STIM protein, and oxidizing ORAI or STIM protein [35, 38]. Studies have shown that ROS regulates the ORAI/STIM channel by directly targeting the conserved cysteine residues in ORAI and STIM molecules [39, 40]. ROS can also act as the second messenger in cells to promote the increase of intracellular Ca^2+^ concentration and lead to vasoconstriction, thus assisting the pathogenesis of hypertension [41]. Ang-II-induced hypertension involves redox-dependent signal cascade activation and NADPH oxidase-induced ROS production [42]. Some common antihypertensive medications, such as Ang-I receptor blockers and angiotensin-converting enzyme (ACE) inhibitors, have been shown to reduce blood pressure partly by inhibiting NADPH oxidase and reducing the ROS production [43].

### 4.2. Atherosclerosis

Atherosclerosis is one of the main causes of cardiovascular death in developed countries [17]. More and more evidence shows that oxidative stress plays a key role in the formation of atherosclerosis [44, 45]. The activation of proinflammatory signal pathway, the expression of cytokines/chemokines, and the increase of oxidative stress are some of the mechanisms underlying atherosclerosis [20].

ROS is an autophagy trigger factor. Excessive ROS in cells is able to cause oxidative stress, which will further activate autophagy [46, 47]. Autophagy is closely related to the development of atherosclerosis [48, 49]. Excessive autophagy can lead to autophagic cell death [50]. Autophagic death of endothelial cells can damage plaque, form thrombus, and cause atherosclerosis [51]. Therefore, elucidating the specific mechanism of ROS-regulating autophagy may be a feasible way to treat atherosclerosis.

**Figure 2:**
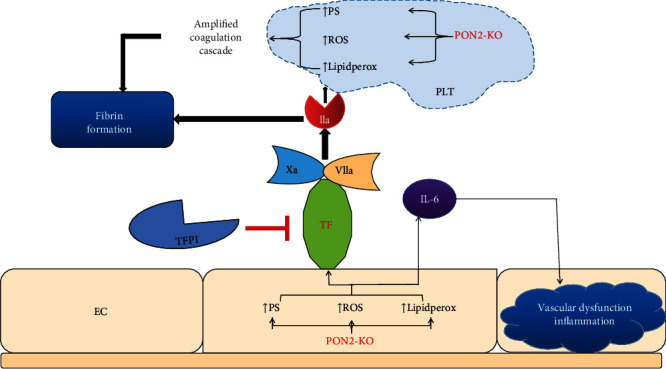
Schematic of EC and platelet-mediated procoagulant and vascular inflammatory processes in pon2^−/−^ environment. EC-mediated systemic inflammation is established by elevated levels of interleukin-6 (IL-6), which may promote vascular inflammation and dysfunction. Knockout of PON2 can lead to the accumulation of phosphatidylserine (PS), ROS, and lipidperox in ECs; result in the production of fibrin through cascade reaction; and ultimately consolidate the function of blood coagulation.

Oxidative stress reduces the expression of prethrombotic antioxidant P-oxidase-2 (PON2) in human atherosclerotic plaques, especially in endothelial cells [52]. Ebert et al. revealed the redox-dependent mechanism of PON2, which involves tissue factor (TF) activity in endothelial cells and prevents systemic coagulation activation and inflammation (Figure 2) [52].

NADPH oxidase is the main source of ROS in atherogenesis, enhancing the production of superoxide and aggravating oxidative stress, leading to the occurrence and development of arterial disease [53, 54]. Gray et al. [55] knocked out the NOX1 and NOX4 genes in streptozotocin (STZ-) induced diabetic ApoE^−/−^ mice and found that loss of NOX1 had a significant antiatherosclerotic effect, which was related to the decreased production of ROS. GPX4 is one of the glutathione peroxidases, which can effectively interact with lipid hydrogen peroxide and catalyze the degradation of peroxides [56]. Mitochondrial GPX4 can avoid ROS damage and maintain intravascular homeostasis by clearing ROS. Overexpression of mitochondrial GPX4 can alleviate myocardial ischemia/reperfusion injury [57]. GPX4 can inhibit ferroptosis by scavenging lipid peroxides and improve the function of the heart [58–60]. GPX4 overexpression inhibits atherosclerosis in ApoE^−/−^ mice [61, 62]. Hyperglycemia can increase the production of ROS, such as O_2_− and peroxide, through the mitochondrial electron transport chain, and then form a positive feedback effect [63, 64]. For example, PKC can be activated by O_2_−. Then, activated PKC can promote the production of NADPH oxidase-dependent ROS [65, 66]. O_2_− in mitochondria can increase the production of intracellular advanced glycation end products (AGEs) in cells [67, 68]. AGEs can add oxygen radical, and the activation of AGEs receptor can cause intracellular oxidative stress, which in turn causes inflammation in endothelial cells [69–71]. Therefore, AGEs eventually lead to atherosclerosis by modifying the extracellular matrix and circulating lipoproteins and activating AGEs receptors [66]. In addition, Zhu et al. showed that AGEs could accelerate vascular calcification through the pathway of hypoxia-inducer/pyruvate dehydrogenase kinase 4 [72].

It is worth mentioning that a recent research found that colchicine, which is a drug widely used in the treatment of nonspecific inflammation, could combine with cholesterol crystal (CC), an important pathological marker for the vulnerability of atherosclerotic plaques [73]. This combination can reduce the intake of cholesterol crystals by endothelial cells, thus attenuating the cellular oxidative stress and endothelial cell prolapse by regulating the AMPK/SIRT1 signaling pathway [74].

### 4.3. Diabetic Cardiomyopathy

The complications of diabetes mainly include nephropathy, neuropathy, retinopathy, and heart diseases, which are linked with the activation of a series of oxidative stress in the body [75]. ROS can interact with a variety of biological macromolecules, such as DNA, proteins, and lipids [76, 77]. In the case of DNA damage, ROS induces DNA strand breaks and the formation of 8-hydroxydeoxyguanosine, which is a prominent feature of the diabetic heart [78]. The passive stiffness of myocardium is redox dependent, which leads to the increase of cardiac stiffness through actin oxidation and disulfide bond formation [79, 80]. In patients with diabetes, oxidative stress leads to decreased actin phosphorylation by damaging the NO/cGMP/PKG signaling [81], increased cardiomyocyte stiffness, and collagen and AGE deposition [82]. Polyunsaturated fatty acids rich in membrane lipids are easily oxidized by ROS, which is also involved in the formation of atherosclerotic plaques [83]. Lipid oxidation can lead to excessive formation of carbonyl compounds, such as aldehydes, which can accelerate a variety of pathologies [84]. NADPH oxidase is the main source of cardiac ROS, in which NOX2 and NOX4 are the two main subtypes expressed in the heart. It has been found that ROS produced by NOX is a common downstream mediator of various hemodynamic and metabolic pathways. ROS is involved in the occurrence of endothelial dysfunction and the development of diabetic vascular complications during hyperglycemia [85]. Glucose autooxidation, PKC activation, GAPDH inhibition, AGE formation, and polyol pathway activation can in turn exacerbate oxidative stress [86–88]. For example, the activation of the PKC pathway can lead to an increased expression of nuclear factor *κ*B(NF-*κ*B) [65, 89]. NF-*κ*B can increase the expression of inducible nitric oxide synthase and increase the production of nitric oxide. Excessive nitric oxide reacts with peroxynitrates to produce peroxynitrates. Peroxynitrates can induce the formation of mitochondrial permeability transition holes, resulting in the increase of ROS production and the loss of cytochrome C, which exacerbates the development of diabetic cardiomyopathy [65]. Related studies found that in the aorta of STZ-induced diabetic ApoE^−/−^ mice, the levels of NOX2 and NOX4 increased; in db/db mice (type II diabetes model), the expression of NOX1 and NOX4 was upregulated, and their activation resulted in the oxidation of ROS downstream molecules (e.g., tetrahydrobiopterin) and increased inflammatory response, indicating that NOX1, NOX2, and NOX4 are all involved in the pathological process of diabetic cardiomyopathy [90].

Of course, hyperglycemia is not the only pathogenic factor of diabetic cardiomyopathy, and the excessive oxidation of free fatty acids is also not ignored, which will lead to the activation of oxidative stress mitochondria and endoplasmic reticulum stress proinflammatory signals [91–93]. What can be seen is that a large number of changes in the diabetic heart include the overexpression of ROS and abnormal redox status. It is believed that the genes or drugs that target to block the ROS pathway in the future will bring the dawn of cure to patients with diabetic cardiomyopathy.

ROS also participates in the cardiac hypertrophy signaling transduction. In insulin-induced cardiac hypertrophy, the ROS level was upregulated and the levels of catalase were decreased [94]. Hypertrophy agonist Ang-II can increase the ROS levels in cardiomyocytes, and mitochondrial oxidative stress in turn contributes to Ang-II-induced cardiac hypertrophy [95]. Antioxidant administration can inhibit cardiac hypertrophy [96]. Tumor necrosis factor-alpha causes hypertrophy via the generation of ROS in cardiomyocytes [97]. Several hypertrophic stimuli need ROS to trigger cardiac hypertrophy. ROS could be a potential biological target for the novel therapy for maladaptive cardiac hypertrophy.

### 4.4. Myocardial Infarction (MI)

MI is one of the leading causes of disability and death in patients with CVDs in the world [98]. Programmed cardiomyocyte death, that is, apoptosis or autophagy, is considered to be the cause of MI. Cardiomyocyte apoptosis induced by ROS is controlled by a complex network of signal pathways involving noncoding RNAs [99]. For instance, under anaerobic conditions, mitochondrial fission and apoptosis-related circRNA (MFACR) suppresses the uninterrupted expression of miR-652-3p and MTP18 proteins, which leads to the imbalance of ROS, triggers the accumulation of mitochondrial fragments, and then results in apoptotic cell death [100]. ROS is involved in the toll-like receptor 4 (TLR4) and its downstream molecular pathway in mediating sympathetic activity post-MI within the paraventricular nucleus (PVN) [101]. The activation of TLR4 enhances the sympathetic activity after myocardial infarction by activating the microglia NF-*κ*B and ROS in the paraventricular nucleus of the hypothalamus.

### 4.5. Heart Failure (HF)

Heart failure (HF) is a progressive disease with an annual mortality rate of about 10%. Although effective treatment has improved the outcome, the prognosis is still poor [102]. Related experiments and clinical studies have shown that the increase of ROS is related to the pathogenesis of HF [103–106]. ROS stimulates myocardial growth, matrix remodeling, and cellular dysfunction by activating various hypertrophic signal kinases and transcription factors. Activation of G protein-coupled receptor (GPCR) can lead to the production of ROS. Some data shows that ROS can directly induce the dissociation and activation of G protein [107–110]. Therefore, ROS may promote the hypertrophic growth signal of neonatal rat ventricular myocytes by directly activating G protein. ROS also stimulates apoptosis signal kinase-1, a redox-sensitive kinase that, when overexpressed, leads to NF-*κ*B-induced hypertrophy [111]. Mitochondrial ROS and mitochondrial matrix calcium ([Ca^2+^]m) also participate in the pathogenesis of obesity-induced heart failure [112, 113], which may attack cardiomyocytes through the mechanism of free radical injury and combined with inflammatory cytokines (such as TNF-*α* and IL-6), resulting in the apoptosis of some cardiomyocytes, decreased cardiac function, and compensatory proliferation of cardiomyocytes, finally leading to myocardial hypertrophy [114].

In vitro hydrogen peroxide treatment induces oxidative stress in cardiomyocyte and leads to all kinds of cellular physiological or pathological processes, including necrosis and apoptosis. Wang et al. had identified several de novo pathways that underlie these processes. Several noncoding RNAs play functional roles in these pathways. In the programmed necrosis induced by hydrogen peroxide, long noncoding RNA NRF can combine with miR-873 and regulate the RIPK1/RIPK3 expression [115]. E2F1/miR-30b/Cyclophilin D forms a pathway in regulating hydrogen peroxide-induced necrotic cell death [116]. During hydrogen peroxide induced apoptosis, Wang et al. found that both E2F1/miR-421/Pink signal pathway and miR-361/PHB1 function in regulating mitochondria fission and apoptosis [118, 119]. All these results indicate that functional noncoding RNAs also play important role in a series of hydrogen peroxide-induced cellular responses. And these studies suggest that there might be relationships between functional noncoding RNAs and ROS, which await further study to unveil.

### 4.6. Atrial Fibrillation (AF)

Atrial fibrillation (AF) is the most common arrhythmia in clinics, and its risk increases with age [119]. Both human and animal data confirm the role of oxidative stress in the pathogenesis of AF [120, 121]. So far, there are some antioxidants that can positively affect the development of AF [122]. Type 2 ryanodine receptor (RyR2) is the main calcium release channel in atrial myocytes. It is a dysfunction caused by oxidative stress which disturbs the intracellular Ca^2+^ homeostasis that is linked with the pathogenesis of AF [123]. In atrial myocytes, RyR2 is oxidized by mitochondrial-derived ROS, resulting in increased intracellular Ca^2+^ leakage. It is worth noting that studies have shown that reducing the production of ROS can reduce atrial diastolic Ca^2+^ leakage, thus hindering the development of AF [124].

### 4.7. DNA Methylation and CVDs

DNA methylation, in which methyl is added to the C-5' position in the dinucleotide sequence of cytidine-phosphate-guanosine (CpG) to inhibit gene activity by preventing transcription factors from binding to the promoter or by recruiting chromatin modifying enzymes [125]. DNA methylation is catalyzed by three different DNA methyltransferases (DNMTs): DNMT3a and DNMT3b are mainly responsible for the ab initio methylation of embryonic and postpartum tissues, while DNMT1 subsequently maintains methylation [126].

The latest advances in next-generation sequencing technology have provided de novo understanding of DNA methylation. And more and more studies found that there are significant contributions of noncoding RNA in the pathophysiology of HF [127].

Long noncoding RNAs (lncRNAs) can regulate gene expression at the epigenetic level by directly or indirectly regulating the interaction with other molecules [128]. lncRNAs show epigenetic characteristics similar to those of coding genes, such as maternal effects, DNA methylation and histone modification, and posttranscriptional regulation [129].

In a series of causes, abnormal gene expression may be related to specific DNA methylation. The specific knockout of DNA methylase DNMT3b gene in the heart can lead to cardiomyocyte interstitial fibrosis and sarcomere disorder and accelerate the deterioration of systolic function and thinning of the ventricular wall during HF [130]. lncRNA-H19 is closely related to genomic imprinting [131]. It can change the methylation level of DNA by regulating the activity of S-adenosyl methionine(SAM), which plays an important role in cardiovascular diseases [129]. lncRNA-Mhrt can directly interact with histone modifiers to regulate chromatin modification, and its upregulated expression can prevent pathological myocardial hypertrophy [128]. lncRNA upperhand can regulate the expression of the hand2 gene related to cardiac development by allele specificity and cis-regulation [132].

The interaction between lncRNA-Chaer and the catalytic subunit of histone modification complex PRC2 interferes with the targeted genomic site of PRC2, thus inhibiting the methylation of histone H3 lysine 27 residues in the promoter region of cardiac hypertrophy related genes [133]. Inhibition of Chaer can significantly reduce myocardial hypertrophy and dysfunction.

The application of the targeted drugs to interfere with epigenetic dynamics is likely to become a new direction of drug research and development for cardiovascular diseases in the future. For example, trichostatin A, a class I and II histone deacetylase (HDACs) inhibitor, can prevent ischemia-induced left ventricular remodeling by inhibiting the TNF-*α* transcription and promote angiogenesis and cardiomyocyte survival by enhancing the Akt phosphorylation [134]. HDAC inhibitor sodium butyrate can block NF-*κ*B signal transduction and inflammatory factors and improve myocardial infarction and atherosclerosis [135]. In addition, folic acid, histone deacetylase inhibitor apicidin, peroxisome proliferator-activated receptor-gamma agonist, and valproic acid were found to contribute to the restoration of chromatin modification in cardiac metabolism [136].

## 5. Diet Participates in the Regulation of ROS

More and more evidences have pointed that diet is closely linked to inflammation. And some researchers have found that it is possible to reduce the incidence of coronary heart disease through controlling diet [137]. If eating high-refined starch, sugar, saturated fatty acids, and trans fatty acids is kept for a long time, it will lead to a lack of natural antioxidants, fibers, and omega-3 fatty acids, which produce excessive proinflammatory cytokines.

In order to explore the relationship between diet and inflammation, Cavicchia et al. [138] proposed the inflammatory diet index (DII) for the first time in 2009, which is a dietary tool derived from the literature to evaluate the overall inflammatory potential of individual diet. DII consists of a variety of dietary ingredients, classified according to proinflammatory and anti-inflammatory components (Table 1). In recent years, DII has been widely used in clinical research, for example, cancer and CVDs [139]. DII can provide new ideas for the diagnosis and treatment of diseases, but related research remains unclear. In the study of diet and CVDs, we need to focus on the huge role of gut microbes and their metabolites in CVDs [140]. There are huge microecosystems in the intestinal tract, in which there are a large number of bacteria, fungi, viruses, protozoa, etc. Its metabolites play an important role in host metabolism, neurodevelopment, energy balance, and immune regulation, as well as the occurrence and development of cardiovascular diseases [141]. For example, intestinal microorganisms can promote vascular dysfunction and hypertension induced by Ang-II through vascular immune cell infiltration and inflammation [142, 143]. In patients with heart failure, the decrease of cardiac output and blood redistribution lead to reduced intestinal perfusion and breakdown of the intestinal barrier, as intestinal microbes and endotoxins enter the bloodstream and increase systemic inflammation, which in turn increases heart failure [144]. The evidence suggests that trimethylamine-*N*-oxide (TMAO) and short-chain fatty acids (SCFAs), the main metabolites of intestinal microorganisms, are involved in the pathogenesis of cardiovascular diseases [145]. TMAO can induce endothelial dysfunction and monocyte adhesion by activating NF-*κ*B, protein kinase C, and pyran domain of nucleotide-binding oligomerization domain-like receptor family, and increase the expression of vascular endothelial inflammatory factors [146, 147]. At the same time, TMAO can also upregulate scavenger receptors in macrophages, promote the accumulation of cholesterol and formation of foam cells in macrophages, and further promote the formation of vascular plaques [148] and promote the inflammatory reaction of blood vessels through the MAPK and NF-*κ*B pathways [149]. SCFAs play a key role in maintaining intestinal barrier function and play a positive role in cardiac metabolic health [150]. In addition, some probiotics and their fermented products have been proved to inhibit the production of nitrogen oxides in macrophages, reduce the types of reactive oxygen species, increase dietary calcium absorption, and thus reduce blood pressure [151].

A dietary intervention has been shown to reduce the risk of cardiovascular disease events. High-fat and high-sugar diets can lead to abnormal intestinal flora and increase the risk of cardiovascular disease [152]. Increasing the carbohydrate diet can change the composition of Rosella and rectal true bacilli [153]. A diet rich in dietary fiber can promote the growth of beneficial bacteria and inhibit the growth of the conditional pathogenic bacteria [154]. A high-fiber diet can increase acetate-producing microorganisms, lower blood pressure, and improve ventricular remodelling and fibrosis [155]. Another example in rats after partial nephrectomy indicated that the use of curcumin in ginger can retain the ejection fraction and reduce the lipid peroxidation of the heart muscle [156]. Allicin (40 mg/kg/day, orally), which is a component of garlic extract, could reduce hypertension, lipid, and protein oxidation in the heart, meanwhile accelerate the levels of antioxidant enzymes [157]. Supplementation of 800 IU/day vitamin E as an antioxidant can reduce CVD endpoints and myocardial infarction in haemodialysis patients with CVDs [158].

Aloe is an edible plant in daily life [159], which contains a compound called aloe-emodin (AE) [160, 161]. Yu et al. [162] found that in the H_2_O_2_-induced apoptosis model of neonatal rat ventricular myocytes, AE can prevent myocardial infarction by upregulating miR-133, inhibiting the ROS production, and inhibiting the caspase-3 apoptosis signal pathway. In addition, AE treatment significantly reversed the H_2_O_2_-induced upregulation of Bax/Bcl-2 and loss of mitochondrial membrane potential. Chen et al. [163] established a rat cardiac inflammation model induced by hyperlipidaemia, and then administered AE to study the potential role and mechanism of AE regulating cardiac oxidative stress and inflammation induced by hyperlipaemia. They found that compared with the normal diet (ND) group, the expression levels of proinflammatory cytokines IL-1*β*, IL-6, and TNF-*α* were significantly upregulated in the hyperlipaemia group, while the expression levels of IL-1, IL-6, and TNF-*α* were dramatically decreased in the AE treatment group. In addition, AE also inhibited the expression of vascular cell adhesion molecule-1 (VCAM1) and intercellular adhesion molecule-1 (ICAM-1). In vitro, AE decreased the expression of IL-1*β*, IL-6, and TNF-*α* in palmitic acid (PA-) treated H9C2 cells in a dose-dependent manner. Further experiments showed that AE inhibited PA-induced cell death and promoted the production of intracellular ROS [163]. This study indicates that AE may reduce cardiac inflammation induced by hyperlipidaemia/plasminogen activator by inhibiting the TLR4/NF-*κ*B signal pathway, which may be a promising therapeutic strategy for the prevention of myocardial injury.

Intestinal flora and cardiovascular disease is a new research field in the future, but the specific mechanism of the interaction between intestinal flora and body is not clear. Maintaining the homeostasis of intestinal flora and correcting the imbalance of intestinal flora will become a new target for the prevention and treatment of cardiovascular diseases.

## 6. Exercise Promotes Balance of ROS

Physical activity has long been considered to be beneficial for CVDs. However, the molecular mechanisms by which triggering and sustaining exercise are beneficial for the heart are poorly understood, which is expected for new therapeutic targets. To explore these mechanisms, Moreira et al. identified cardiac gene targets in rat models by using RNA sequencing [164], whose expression could be disrupted in heart failure but was recovered by exercise. Through a series of elaborate validation, they screened 16 targets to assess whether targeted interference with the silencing RNA of these genes can affect the abundance of a CVD biomarker (BNP, B-type natriuretic peptide) in human cardiomyocytes. Among them, the Proline Dehydrogenase (PRODH) expression is reduced in human failing hearts, but rescued by exercise in a rat model of HF. The knockdown of PRODH also resulted in the rise of the BNP expression in human cardiomyocytes.

Compared with the traditional drug treatments, natural methods of improving the collaterals through exercise training seem to be more effective, especially for patients with intermittent claudication. Exercise has a variety of positive effects on the body, but it also has systemic benefits [165, 166]. In general, physical activity has been shown to greatly improve cardiovascular function, and this is partly due to improved bioavailability of NO, increased endogenous antioxidant defence, and decreased expression of the enzyme involved in the ROS production [167].

## 7. Summary

**Figure 3:**
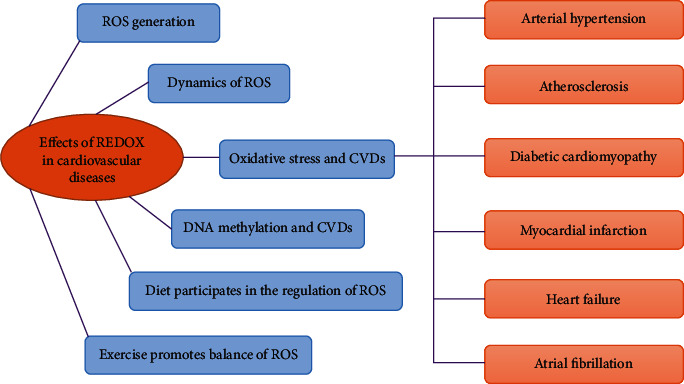
Summary exhibiting effects of REDOX in cardiovascular disease. ROS plays an important role in cardiovascular disease and serious heart disease. The pathogenesis of cardiovascular diseases and metabolic diseases is complex, and understanding the tissue-specific REDOX signal is very important for us to develop new methods to treat diseases. The contribution and influence of reactive oxygen species on metabolic processes is an important area of scientific discovery. Exploring the regulatory effects of a healthy diet and exercise on the improvement of oxidative stress, inflammation, and cardiac dysfunction will also provide new directions for the treatment of CVD.

ROS is not only a natural by-product of metabolic responses in various cell compartments but also a signalling molecule that regulates specific biochemical pathways in normal cell function and survival. However, the dysregulation of ROS signalling or excessive production of nonspecific ROS can affect the pathophysiology of heart diseases. As this review highlights (Figure 3), ROS are particularly important in cellular metabolism and inflammatory signalling. Therefore, it is not surprising that ROS plays an important role in cardiac diseases associated with metabolic disorders and inflammation. The pathogenesis of cardiovascular and metabolic diseases is complex, and understanding tissue-specific REDOX signals is important for us to develop new and novel therapies to treat diseases. Metabolic dysregulation is a major driver of cell dysfunction and disease progression, and exploring the contribution and effect of reactive oxygen species on metabolic processes is an important field of scientific discovery. At the same time, exploring the effects of a healthy diet and exercise on the regulation of oxidative stress, inflammation, and the improvement of cardiac dysfunction will also provide a new direction for the treatment of CVDs.

## Figures and Tables

**Table 1:** Components of the inflammatory dietary index.

Name	Proinflammatory or anti-inflammatory	Total inflammatory score
Alcohol (g)	Anti-inflammatory	-0.278
Anthocyanidins (mg)	Anti-inflammatory	-0.131
Beta carotene (*μ*g)	Anti-inflammatory	-0.584
Black/green tea (g)	Anti-inflammatory	-0.536
Caffeine (g)	Anti-inflammatory	-0.110
Carbohydrate (g)	Proinflammatory	0.097
Cholesterol (mg)	Proinflammatory	0.110
Energy (kcal)	Proinflammatory	0.180
Eugenol (mg)	Anti-inflammatory	-0.140
Fiber (g)	Anti-inflammatory	-0.663
Flavan-3-ol (mg)	Anti-inflammatory	-0.415
Flavonols (mg)	Anti-inflammatory	-0.467
Folic acid (*μ*g)	Anti-inflammatory	-0.190
Garlic (g)	Anti-inflammatory	-0.412
Ginger (g)	Anti-inflammatory	-0.453
Iron (mg)	Proinflammatory	0.032
Isoflavones (mg)	Anti-inflammatory	-0.593
Magnesium (mg)	Anti-inflammatory	-0.484
Monounsaturated fatty acids (g)	Anti-inflammatory	-0.009
Niacin (g)	Anti-inflammatory	-0.246
Omega 3 (g)	Anti-inflammatory	-0.436
Omega 6 (g)	Anti-inflammatory	-0.159
Onion (g)	Anti-inflammatory	-0.301
Oregano/thyme (mg)	Anti-inflammatory	-0.102
Pepper (g)	Anti-inflammatory	-0.131
Polyunsaturated fatty acids (g)	Anti-inflammatory	-0.337
Protein (g)	Proinflammatory	0.021
Riboflavin (mg)	Anti-inflammatory	-0.068
Rosemary (mg)	Anti-inflammatory	-0.013
Saturated fat (g)	Proinflammatory	0.373
Selenium (*μ*g)	Anti-inflammatory	-0.191
Thiamine (mg)	Anti-inflammatory	-0.098
Total fat (g)	Proinflammatory	0.298
Trans fat (g)	Proinflammatory	0.229
Turmeric (mg)	Anti-inflammatory	-0.785
Vitamin A (RE)	Anti-inflammatory	-0.401
Vitamin B6 (mg)	Anti-inflammatory	-0.365
Vitamin B12 (*μ*g)	Proinflammatory	0.106
Vitamin C (mg)	Anti-inflammatory	-0.424
Vitamin D (*μ*g)	Anti-inflammatory	-0.446
Vitamin E (mg)	Anti-inflammatory	-0.419
Zinc (mg)	Anti-inflammatory	-0.313
